# Electrospun 5-chloro-8-hydroxyquinoline-Loaded Cellulose Acetate/Polyethylene Glycol Antifungal Membranes Against Esca

**DOI:** 10.3390/polym11101617

**Published:** 2019-10-05

**Authors:** Mariya Spasova, Nevena Manolova, Iliya Rashkov, Mladen Naydenov

**Affiliations:** 1 Laboratory of Bioactive Polymers, Institute of Polymers, Bulgarian Academy of Sciences, Acad. G. Bonchev St, bl. 103A, BG-1113 Sofia, Bulgaria; rashkov@polymer.bas.bg; 2 Department of Microbiology, Agricultural University, BG-4000 Plovdiv, Bulgaria; mladen@au-plovdiv.bg

**Keywords:** electrospinning, cellulose acetate, polyethylene glycol, 5-chloro-8-hydroxyquinoline, *Phaeomoniella chlamydospora*, *Phaeoacremonium aleophilum*, esca

## Abstract

Esca is one of the earliest described diseases in grapevines and causes trunk damage and the sudden wilting of the entire plant; it is caused mainly by the species *Phaeomoniella chlamydospora* (*P. chlamydospora*) and *Phaeoacremonium aleophilum* (*P. aleophilum*). In practice, there are no known curative approaches for fighting esca directly, which is a huge problem for preserving vineyards. Micro- and nanofibrous membranes from cellulose acetate (CA) and cellulose acetate/polyethylene glycol (CA/PEG) containing 5-chloro-8-hydroxyquinolinol (5-Cl8Q) were successfully prepared by electrospinning. The surface morphologies and optical and mechanical properties of the membranes were characterized by using scanning electron microscopy (SEM), Fourier transform infrared spectroscopy (FTIR), ultraviolet-visible spectroscopy (UV-Vis), water contact angle measurements and mechanical tests. It was found that the bioactive compound release was facilitated by PEG. The antifungal activities of the obtained materials against *P. chlamydospora* and *P. aleophilum* were studied. We have demonstrated that 5-Cl8Q is an efficient and sustainable antifungal agent against *P. chlamydospora* and *P. aleophilum*. Moreover, for the first time, the present study reveals the possibility of using electrospun polymer membranes containing 5-Cl8Q which impede the penetration and growth of *P. chlamydospora* and *P. aleophilum*. Thus, the obtained fibrous materials can be suitable candidates for plant protection against diverse fungi.

## 1. Introduction

Electrospinning is currently regarded as one of the most promising nanotechnologies for the preparation of fibers with micro- and nanoscale diameters and a large specific surface area, which is a prerequisite for the attainment of high effectiveness in a number of applications; e.g., medicine and agriculture [1,2].

Esca is grapevine trunk disease that causes dark red or yellow stripes on leaves, trunk damage and the sudden wilting of the entire plant [3,4]. It is known that esca disease is caused mainly by the species *Phaeomoniella chlamydospora* and *Phaeoacremonium aleophilum* [5,6]. Over the last three decades, the impact of esca disease has become a dramatic global threat to all vineries. The wounds formed during the pruning procedure on vines are considered to be one of the main entrances for the penetration of *Phaeomoniella chlamydospora* and *Phaeoacremonium aleophilum* spores in grapevines. To date, sodium arsenite is the only known and effective agent for fighting esca; however, arsenic and its compounds have recently been classified as carcinogenic and their use has been disallowed. Thus, in practice, there are no known curative approaches for fighting esca directly. This requires the development of new approaches to fight esca in order to prevent the loss of vineyards and losses for the wine industry.

Materials consisting of rayon membranes on which electrospun nanofibers of soy protein/polyvinyl alcohol and soy protein/polycaprolactone are deposited have been proposed for physically blocking fungal spore penetration [7]. It has been reported that the physical blocking is insufficient, and the inclusion of an antifungal component has been put forward [8]. The proposed materials consist of lactide/glycolide copolymer and poly(butyleneadipate-*co*-terephthalate) blended with a specially synthesized antifungal polymer–polyhexamethylene guanidine. In our study, we propose the facile preparation of fibrous membranes containing an easily available and efficient antifungal compound, 8-hydroxyquinoline derivative, for active protection against spore penetration and plant infection. Moreover, the electrospun micro- and nanostructured membranes allow air and moisture permeability and allow the plant wound to “breathe”.

8-Hydroxyquinoline and its derivatives manifest antibacterial and antifungal activities [9,10] and are of low toxicity to humans [11]. Moreover, we have recently shown that the combination of water-soluble polymers and 8-hydroxyquinoline derivatives results in the obtaining of a stable solutions with antifungal activity suitable for applications in agriculture. The minimum inhibitory concentration (MIC) values of 5-Cl8Q against *P. chlamydospora* and *P. aleophilum* were found to be 0.75 μg/mL for both strains [12].

Cellulose acetate (CA) is one of the most important esters of cellulose. The advantages of CA are its low cost, easily feasible production and wide variety of applications [13]. Recently, great attention has been paid to fibers from cellulose and cellulose derivatives due to their biodegradability and good mechanical and barrier properties [14].

In the present study, a suitable combination of selected polymers (cellulose acetate and polyethylene glycol) with 5-Cl8Q is suggested by using an effective method—electrospinning—that will enable the creation of fibrous polymer membranes with antifungal activity.

The obtained materials were characterized by SEM, FTIR, UV-Vis, water contact angle measurements and mechanical tests. The possibility of modulating the 5-Cl8Q release profile by the appropriate selection of the composition of the polymer matrix was shown. The effect of the fibrous membranes on the biological behavior upon contact with *P. chlamydospora* and *P. aleophilum* was also assessed.

## 2. Materials and Methods

### 2.1. Materials

Cellulose acetate (CA, Aldrich, St. Louis, MO, USA) with ${\bar{M}}_{n}$ at 30,000 g/mol and 39.8 wt% of degree of substitution in acetyl content, polyethylene glycol (PEG, Fluka, Buchs, Switzerland) with relative molecular mass (Mr) 1900–2000 g/mol, polyethylene oxide (PEO, Serva, Heidelberg, Germany) with Mr ca. 100,000 g/mol and 5-chloro-8-hydroxyquinolinol (5-Cl8Q, Sigma-Aldrich, Buchs, Switzerland) were used. Acetone (Sigma-Aldrich) of analytical grade of purity was used. Potato dextrose agar medium was purchased from Merck, Darmstadt, Germany. The disposable consumables were supplied by Orange Scientific, Braine-l’Alleud, Belgium.

### 2.2. Preparation of Fibrous Membranes by Electrospinning

Four types of fibrous membranes were prepared by electrospinning: Type 1: CA (control); type 2: CA/5-Cl8Q, Type 3: CA/PEG (control); and Type 4: CA/PEG/5-Cl8Q. For the preparation of membranes, the following spinning solutions were prepared in acetone/water at 80/20 *v*/*v*: (*i*) CA; (*ii*) CA/5-Cl8Q; (*iii*) CA/PEG; and (*iv*) CA/PEG/5-Cl8Q with a total polymer concentration of 10 wt% and 5-Cl8Q 10 wt.% with respect to total polymer weight.

The electrospinning set-up was composed of a custom-made high-voltage power supply (up to 30 kV), a grounded rotating drum collector, an infusion pump (NE-300 Just InfusionTM Syringe Pump, New Era Pump Systems Inc., New York, NY, USA) to deliver the spinning solution at a constant rate and a syringe equipped with a metal needle (gauge: 20GX1½″). Electrospinning was performed under the following conditions: a flow rate of 3.0 mL/h, voltage of 25 kV, needle tip-to-collector distance of 15 cm, collector rotating speed of 1000 rpm, a room temperature of 21 °C and a relative humidity of 50%.

### 2.3. Characterization of the Fibrous Membranes

The dynamic viscosity of the spinning solutions was measured using a Brookfield DV-II+ Pro programmable viscometer for the cone/plate option equipped with a sample thermostated cup and a cone spindle at 25 ± 0.1 °C.

The morphology of the fibrous membranes was analyzed by SEM. The samples were vacuum-coated with gold and observed by a Jeol JSM-5510 SEM (Tokyo, Japan). The average fiber diameter was estimated by ImageJ software [15] by measuring at least 20 fibers from three different SEM micrographs for a total of 60 measurements, and their morphology was assessed, applying the criteria for the overall evaluation of electrospun materials as described in detail in [16].

Attenuated total reflection Fourier-transform infrared (ATR-FTIR) spectra were recorded using an IRAffinity-1 spectrophotometer (Shimadzu Co., Kyoto, Japan) equipped with a MIRacle™ATR (diamond crystal, depth of penetration of the IR beam into the sample – about 2 μm) accessory (PIKE Technologies, Madison, WI, USA) in the range of 600–4000 cm^−1^ with a resolution of 4 cm^−1^. All spectra were corrected for H_2_O and CO_2_ using an IRsolution software program.

The static contact angle measurements of the membranes were taken using an Easy Drop DSA20E Krüss GmbH drop shape analysis system (Hamburg, Germany) at 20 ± 0.2 °C. A sessile drop of deionized water with a volume of 10 μL controlled by a computer dosing system was deposited onto the membranes (2 cm × 7 cm; cut in the direction of rotation of the collector). The contact angles were calculated by computer analysis of the acquired images of the droplet. The data were averaged from 20 measurements for each sample.

Mechanical properties were evaluated by tensile measurements performed on the fibrous membranes using a single column system for mechanical testing, INSTRON 3344, equipped with a loading cell 50 N and Bluehill universal software. The stretching rate was 10 mm/min, the initial length between the clamps was 40 mm and the room temperature was 21 °C. All samples were cut in the direction of collector rotation with dimensions of 20 × 60 mm and a thickness of ca. 200 µm. For the sake of statistical significance 10 specimens of each sample were tested, after which the average values of Young’s modulus, the ultimate stress and the maximum deformation at break were determined.

The 5-Cl8Q release was studied in vitro at 37 °C in acetate buffer (CH_3_COONa/CH_3_COOH) containing lactic acid (acetate buffer/lactic acid = 96/4 *v*/*v*) at pH 3 and an ionic strength of 0.1. 5-Cl8Q-containing nanofibrous membranes (4 mg) were immersed in 100 mL of buffer solution under stirring in a water bath (Julabo, Germany). The release kinetics was determined by withdrawing aliquots (2 mL) from the solution at determined time intervals, adding back the same amount of fresh buffer and recording the absorbance of the aliquots by a DU 800 UV–vis spectrophotometer (Beckman Coulter) at a wavelength of 255 nm. The amount of released 5-Cl8Q was calculated using calibration curves (correlation coefficient R = 0.999) for the membranes in acetate buffer/lactic acid = 96/4 v/v, pH = 3, at an ionic strength of 0.1. The data are average values from three measurements.

### 2.4. In Vitro Antifungal Assay

The antifungal activity of membranes was monitored against the fungi *P. chlamydospora* Centraalbureau voor Schimmelcultures (CBS) 239.74 and *P. aleophilum* CBS 631.94. *P. chlamydospora* CBS 239.74 and *P. aleophilum* CBS 631.94 were purchased from Westerdijk Fungal Biodiversity Institute, Utrecht, the Netherlands.

There are data in the literature showing that, under laboratory conditions, *P. chlamydospora* and *P. aleophilum* grow normally on potato dextrose agar [17] and malt extract agar [18]. In order to measure the zones of inhibition, in vitro studies were performed using potato dextrose agar medium (PDA, Merck, Darmstadt, Germany) for the fungal strains. The surface of the solid agar was inoculated with a suspension of fungi culture with a fungi concentration of 1 × 10^5^ cells/mL, and on the surface of the agar in each Petri dish, one membrane was placed. The Petri dishes were incubated for 96 h for the *P. chlamydospora* and *P. aleophilum* at 28 °C, and subsequently the zones of inhibition around the disks were measured. The average diameters of the zones of inhibition were determined using the ImageJ software based on 15 measurements in 15 different directions for each zone.

## 3. Results

### 3.1. Preparation of Fibrous Membranes by Electrospinning

In our previous study, the optimal conditions for the electrospinning of CA solutions were found to lead to controllable CA fiber morphologies [19]. However, it is necessary to explore the effect of electrospinning parameters on the morphologies of CA/PEG fibers, offering the potential to controllably produce electrospun CA/PEG fibers as well.

It is well known that the viscosity of the spinning solutions has a significant influence on the electrospinning process and the resultant fiber morphology. Therefore, the dynamic viscosity of the prepared solutions in this study was measured. The viscosities of 10 wt.% solutions of CA (one component), CA/5-Cl8Q (two components), CA/PEG (two components) and CA/PEG/5-Cl8Q (three components) were 184, 190, 91, and 108 cP, respectively.

**Figure 1:**
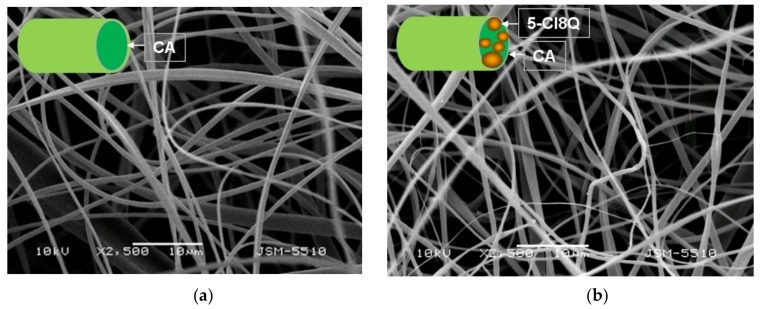

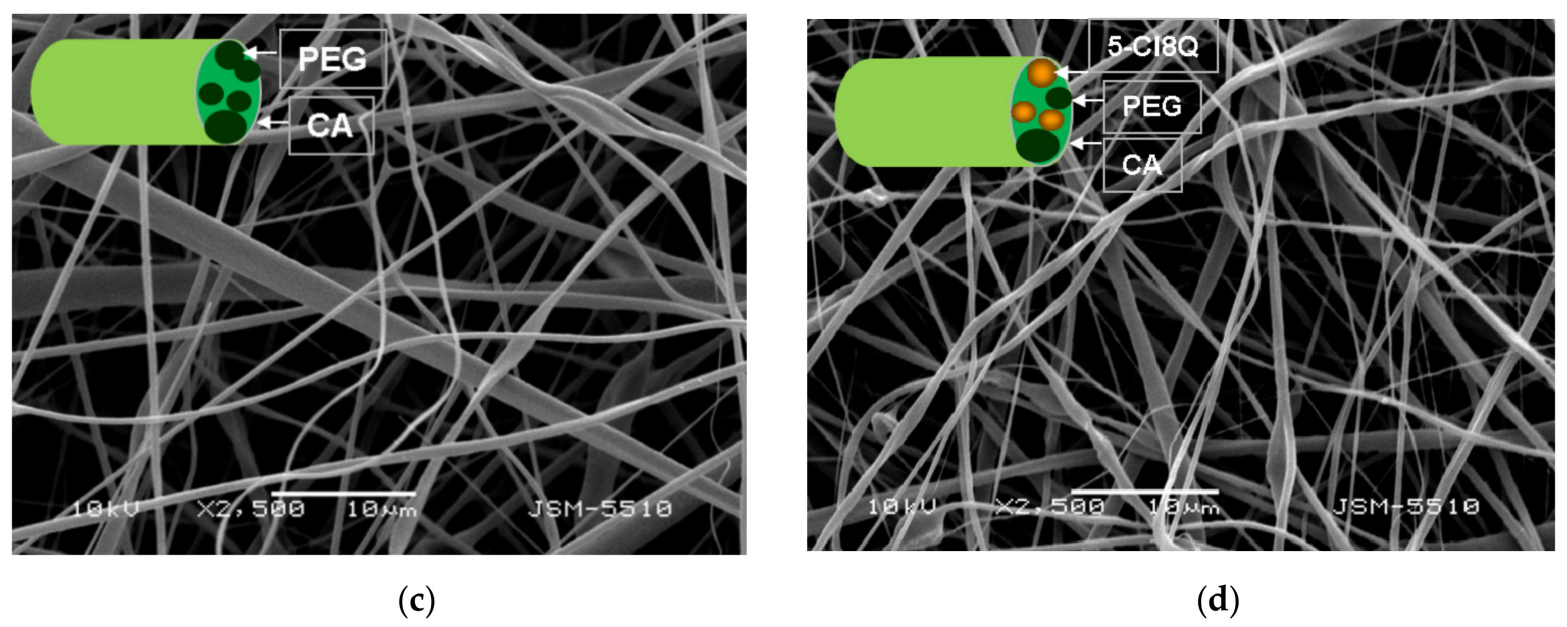
SEM micrographs and schematic representation of the cross-section of fibers (insets) of electrospun membranes of cellulose acetate (CA) (**a**), CA/5-chloro-8-hydroxyquinolinol (5-Cl8Q) (**b**), CA/polyethylene glycol (PEG) (**c**) and CA/PEG/5-Cl8Q (**d**); magnification ×2500.

SEM micrographs of the prepared CA, CA/5-Cl8Q, CA/PEG and CA/PEG/5-Cl8Q membranes are shown in Figure 1. A schematic representation of the cross-section of the fibers constituting the four types of fibrous membranes (CA, CA/5-Cl8Q, CA/PEG and CA/PEG/5-Cl8Q) prepared in the present study is shown in Figure 1 (insets). The electrospinning of CA solution under the selected conditions reproducibly resulted in continuous defect-free fibers with a mean fiber diameter of 780 ± 100 nm (Figure 1a). The addition of PEG into the spinning solutions resulted in a decrease of the dynamic viscosity and decrease of the average fiber diameter. The average diameter of the CA/PEG nanofibers (531 ± 80 nm) was smaller (Figure 1b) than that of the CA nanofibers under constant total polymer concentration and spinning conditions.

Representative SEM images of the obtained CA and CA/PEG membranes containing 5-Cl8Q are shown in Figure 1b,d. The addition of 5-Cl8Q (10 wt%) to the spinning solutions led to the preparation of fibers with smaller diameters of 750 ± 90 nm for the CA/5-Cl8Q fibrous membrane and 446 ± 60 nm for the CA/PEG/5-Cl8Q membrane.

### 3.2. FTIR Spectra of the Fibrous Membranes

**Figure 2:**
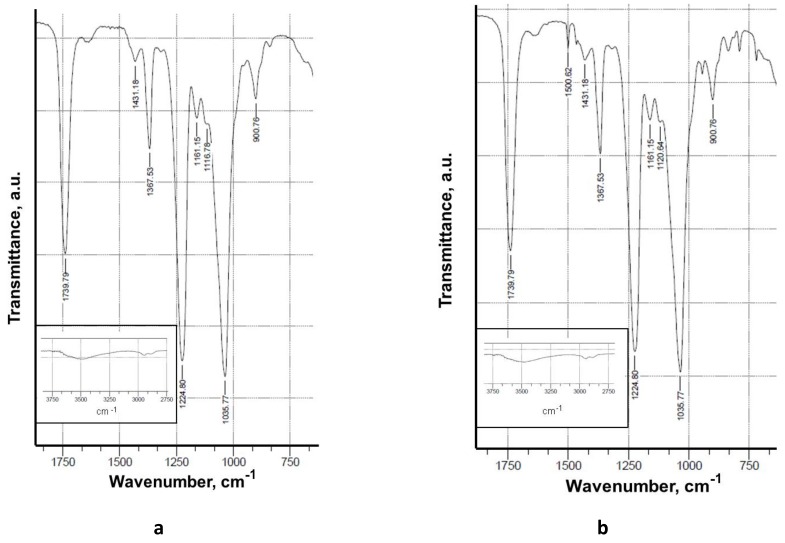
Fourier transform infrared spectroscopy (FTIR) spectra of electrospun membranes of (**a**) CA and (**b**) CA/5-Cl8Q.

The CA and CA/5-Cl8Q fibrous membranes were characterized by FTIR spectroscopy (Figure 2). In the IR spectrum of the CA membrane (Figure 2a), bands characteristic of CA appeared at 1740 cm^−1^ for the C=O groups, at 1369 and 1226 cm^−1^ for the CH_3_ groups, and at 1037 cm^−1^ for the ether C–O–C groups, in accordance with the literature [20]. In the FTIR spectra of CA/5-Cl8Q (Figure 2b) membranes, in addition to the characteristic bands of CA, a new band appeared at 1500 cm^−1^ characteristic of a quinoline ring [21], proving the presence of the bioactive compound in the electrospun membrane. In the spectra of the CA/PEG and CA/PEG/5-Cl8Q, new characteristic bands were observed at 1100 cm^−1^, assigned to the C-O-C ether groups of PEG, as well as at 2875 cm^−1^ characteristic for the vC–H vibrations. In the FTIR spectra of CA/PEG/5-Cl8Q, in addition to the bands characteristic of CA and PEG, new bands appeared at 1500 cm^−1^ which were characteristic of the aromatic ring of the bioactive compound, demonstrating the successful incorporation of the 5-Cl8Q in the CA/PEG membrane.

### 3.3. Water Contact Angle of the Fibrous Membranes

The adhesion of fungal plant pathogens is affected by the hydrophilic/hydrophobic balance of the host surface [22]. Therefore, it is crucial to measure the water contact angle of the obtained membranes that will be in contact with fungal species.

It is well known that when a water drop is placed on a solid surface, it will spread on the surface based on the intermolecular interactions between the solid and the liquid. The water contact angle will immediately give an indication of the wettability of the solid.

**Figure 3:**
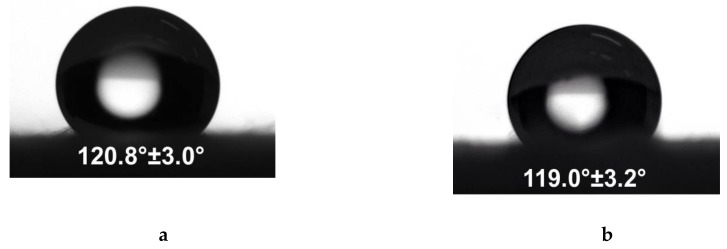

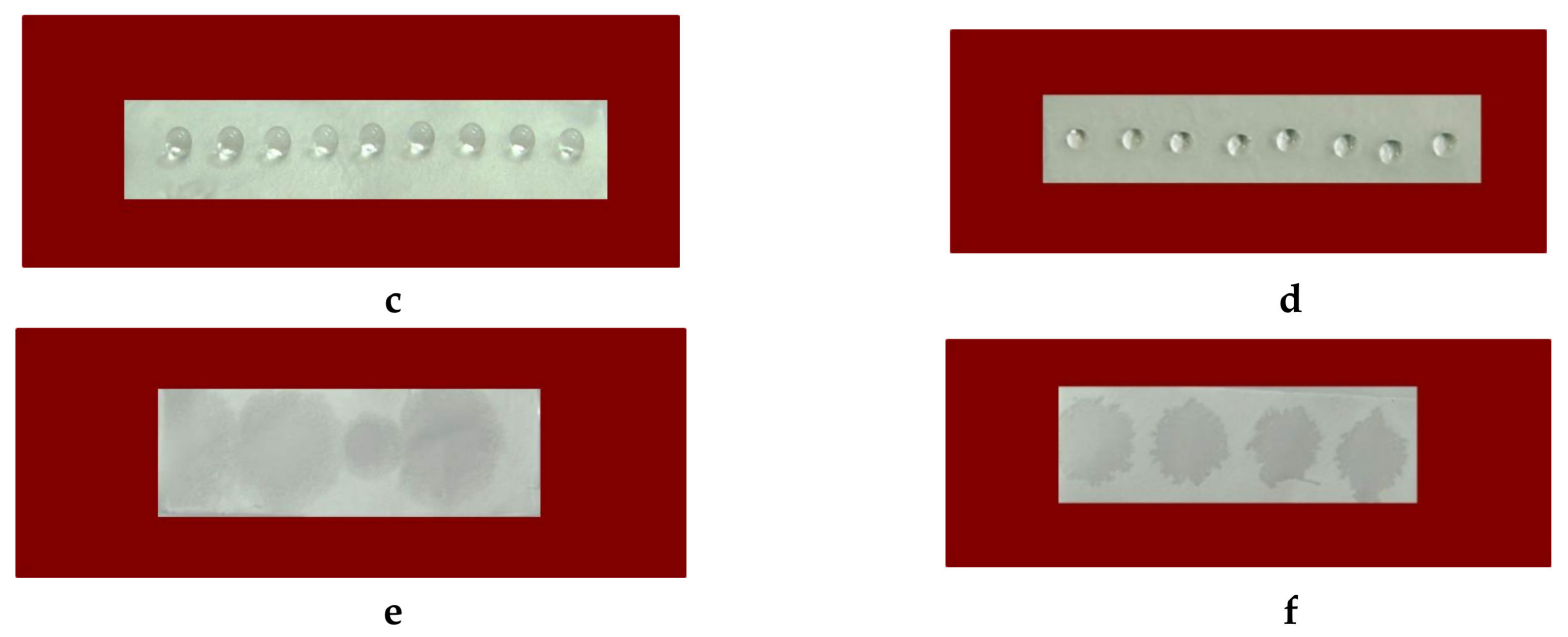
Digital images of water droplets (10 µl) deposited on the membrane surfaces and contact angle values for membranes from (**a**) and (**c**) CA, (**b**) and (**d**) CA/5-Cl8Q, (**e**) CA/PEG and (**f**) CA/PEG/5-Cl8Q.

It was found that the contact angle values depended on the composition of the prepared study membranes. Neat CA fibrous membranes were hydrophobic, with a water contact angle of 120.8° ± 3.0° (Figure 3a,c), and the water droplet preserved its spherical shape within 2 h. The measured contact angle values of CA/5-Cl8Q membranes were 119.0 ± 3.2° and were close to those measured for the CA membranes (Figure 3b,d).

The incorporation of the water-soluble polymer PEG resulted in a significant decrease of the measured contact angle value. The values of the water contact angle of CA/PEG and CA/PEG/5-Cl8Q membranes were 0°, thus indicating complete wetting (Figure 3e,f).

### 3.4. Mechanical Behavior of the Fibrous Membranes

**Figure 4:**
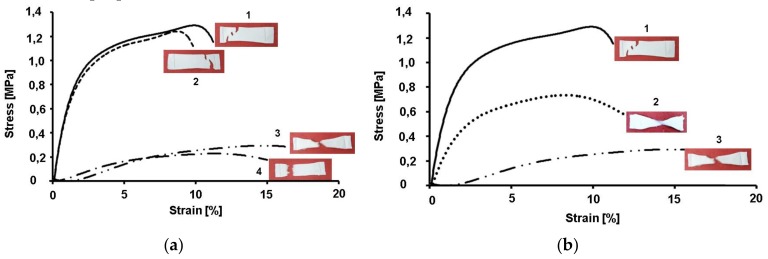
Stress–strain curves of membranes: (**a**) 1. CA, 2. CA/5-Cl8Q, 3. CA/PEG and 4. CA/PEG/5-Cl8Q; (**b**) 1. CA, 2. CA/ PEO_100,000_ and 3. CA/PEG_2000_.

Typical stress–strain curves of CA, CA/5-Cl8Q, CA/PEG and CA/PEG/5-Cl8Q fibrous membranes are shown in Figure 4a. The CA membranes had the highest values of tensile strength. The obtained values are in good agreement with the literature data [23]. The determination of the mechanical characteristics of the CA/5-Cl8Q membranes shows that these materials manifest mechanical properties close to those of CA membranes. This result indicates that the incorporation of the 5-Cl8Q in the fibrous membranes does not considerably alter the mechanical characteristics of the obtained materials. Contrary, it is clearly seen that the addition of PEG—a polymer of low molecular weight—led to a significant decrease in the mechanical properties of the fibrous membranes. The tensile strength of the CA/PEG membrane was ca. 0.2 MPa, while the tensile strength of the CA membrane reached 1.34 MPa.

An original approach to improving the mechanical properties of fibrous materials containing PEG is the use of a polymer with higher molecular weight. This hypothesis was proved using PEO with a molecular weight of 100,000 g/mol for the preparation of CA/PEO_100,000_ membranes by electrospinning and to test their mechanical strength. The stress–strain curves of CA, CA/PEO_100,000_ and CA/PEG_2000_ is shown in Figure 4b. It can be seen that the tensile strength of the CA/PEO_100,000_ membrane reached 0.73 MPa while the tensile strength of the CA/PEG_2 000_ membrane was barely 0.2 MPa. This is evidence that the use of a PEO with higher molecular weight may improve the mechanical properties of the membranes.

### 3.5. In Vitro Release of 5-Cl8Q from the Membranes

**Figure 5:**
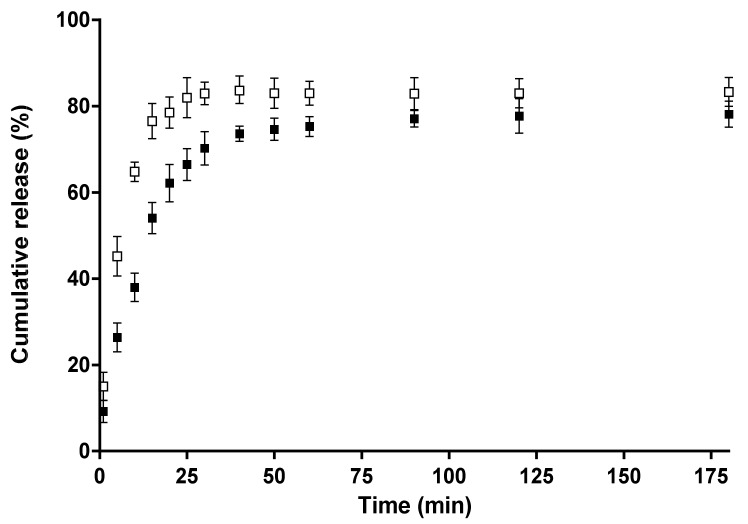
Release profiles of 5-Cl8Q from fibrous membranes of CA/5-Cl8Q (■) and CA/PEG/5-Cl8Q (□). The results are presented as average values from three separate measurements with the respective standard deviation; acetate buffer/lactic acid (96/4 *v*/*v*), pH 3, 37 °C, and an ionic strength of 0.1.

The release of 5-Cl8Q from CA/5-Cl8Q and CA/PEG/5-Cl8Q fibrous membranes was studied spectrophotometrically, and the results are presented in Figure 5. For all membranes, a burst release was initially observed followed by a second stage of gradual release. As seen in Figure 5, the 5-Cl8Q was released slowly from the CA/5-Cl8Q membrane. This observation may be explained by the hydrophobicity of this membrane (a water contact angle value of 120°). The amount of released 5-Cl8Q from the hydrophobic CA/5-Cl8Q membrane was ca. 78% for 175 min. A more rapid release of 5-Cl8Q, and at the greatest amount, stemmed from the CA/PEG/5-Cl8Q membrane. In the case of the CA/PEG/5-Cl8Q membrane, ca. 83% of the loaded amount was released within 30 min. This rapid release of the bioactive compound is most likely due to the higher wettability of the CA/PEG/5-Cl8Q membrane due to the presence of water-soluble polymer PEG in the membrane. Thus, the penetration of the aqueous medium in the membrane and the release of 5-Cl8Q were favored.

### 3.6. Antifungal Activity of the Fibrous Membranes

**Figure 6:**
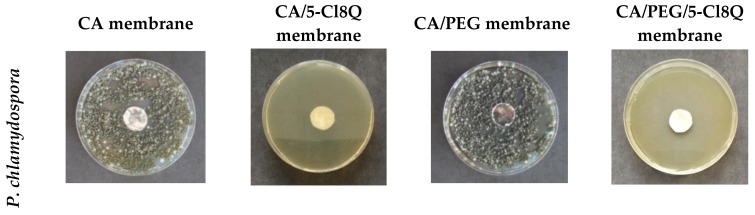

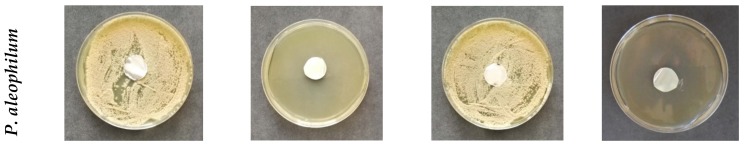
Digital images of the zones of inhibition against *P. chlamydospora* and *P. aleophilum* after the contact of the membranes with fungi cells. The membrane type is indicated at the top of each column. The cell type is marked on the left of each row.

It is known that esca disease is caused mainly by the species *P. chlamydospora* and *P. aleophilum.* In the present study, the antifungal activity of the electrospun membranes was assessed by performing tests against *P. chlamydospora* and *P. aleophilum*. The results obtained by the determination of the zones of inhibition after the contact of the fibrous materials with the fungal cells are shown in Figure 6.

The incorporation of 5-Cl8Q in membranes that were placed in contact with *P. chlamydospora* and *P. aleophilum* resulted in complete inhibition for all fungi. In contrast, as expected, the neat CA and CA/PEG membranes did not alter the fungal growth and did not exhibit any antifungal activity. The observation of wide zones of inhibition around all membranes containing 5-Cl8Q is evidence that the incorporated bioactive compound imparts antifungal activity to the prepared novel fibrous membranes.

## 4. Discussion

**Figure 7:**
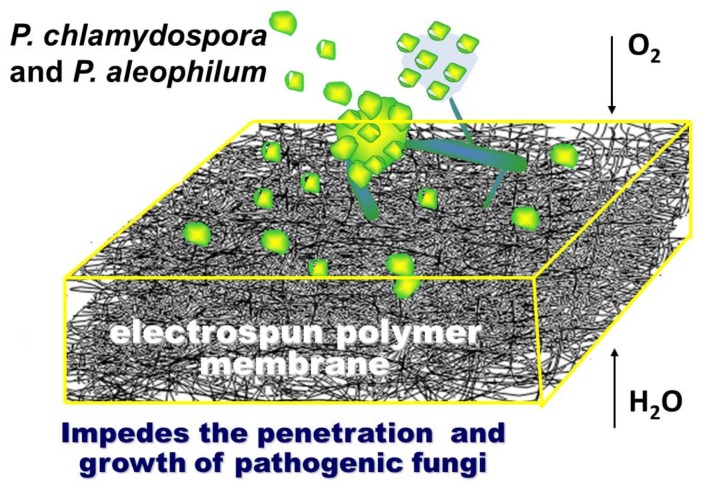
Schematic representation of the research concept.

The schematic representation of the concept of this work is shown in Figure 7. The research concept is based on the assumption that, by using the electrospinning method, it is possible to find an effective experimental approach to obtain innovative micro- and nanostructured polymer membranes with fungicidal activity against *P. chlamydospora* and *P. aleophilum*. Moreover, the so-called “active dressing” made of electrospun polymer membranes will not only be advantageous due to its fungicidal activity against some of esca‘s causes, but it will also allow the plant wound to “breathe”.

Combining the natural polymer CA and water-soluble polymer PEG in materials designed for biomedical and agricultural applications has attracted attention because CA and PEG are polymers which have low toxicity. Moreover, the incorporation of 5-Cl8Q would impart an antifungal activity to the prepared materials.

In the present study, the versatility of electrospinning was exploited in order to create innovative polymer membranes with fungicidal activity against two strains or ascomycete fungi—*P. chlamydospora* and *P. aleophilum*—associated with esca, which is the most devastating grapevine disease.

The study is focused on finding conditions for combining the properties of the polysaccharide derivative cellulose acetate [24] and the polyethers PEG or PEO with a biologically active compound (5-Cl8Q). The incorporation of 5-Cl8Q in the polymer matrix is expected to impart antifungal activity to the obtained membranes. Moreover, PEG and PEO have been reported as solubilizing agents for poorly water-soluble drugs [25] and will lead to the more rapid release of the biologically active compound.

The measurements of the spinning solution viscosity showed that the incorporation of PEG into the CA solution resulted in a viscosity decrease, which was due to the low molecular weight of this polymer. The incorporation of 5-Cl8Q in the solutions of CA and CA/PEG led to insignificant increases in dynamic viscosity values.

In the present study, we used SEM analysis to observe the morphology of the obtained membranes. SEM micrographs of the obtained electrospun CA fibers showed that they were defect-free. The incorporation of PEG in CA resulted in a decrease of the diameters of the obtained fibers compared to neat CA fibers. The observed effect may be explained by the decrease in the solution viscosity by adding a lower-molecular-weight polymer (PEG) to the spinning solution. Moreover, the addition of 5-Cl8Q to the spinning solutions resulted in a slight decrease of the fiber diameter; this decrease is in accordance with previous findings about fibrous materials prepared by the electrospinning of polymer solutions containing an ionogenic low-molecular-weight compound [26,27].

Fourier transform infrared spectroscopy (FTIR) is a reliable analytical tool for the identification of polymers and polymer samples and the quantification of components in polymer mixtures. In the present study, FTIR verified the presence of 5-Cl8Q in the electrospun membranes by the appearance of a new band characteristic of the quinoline ring.

Contact angle analysis was used to give an indication of the surface wettability depending on the composition of the prepared membranes. It was found that CA and CA/5-Cl8Q membranes were hydrophobic. The obtained values of the water contact angle of CA are in good agreement with the literature. There are reports revealing that CA electrospun fibers are hydrophobic and even superhydrophobic [28]. This is explained by the increased surface roughness through electrospinning and the reduction of the surface energy after electrospinning. In the present study, a high contact angle for the CA/5-Cl8Q membrane was determined, indicating that the bioactive compound does not alter the contact angle values and that these membranes remain hydrophobic. This was expected because of the aromatic character of the bioactive compound and the presence of chlorine in its structure.

The most commonly used and feasible method for modulating the hydrophilic/hydrophobic surface properties is the blending of hydrophilic polymers with hydrophobic polymers [29]. In the present study, we incorporate PEG to CA and CA/5-Cl8Q by blending in order to impart hydrophilicity and to ensure a more rapid release of the bioactive compound. As expected, the presence of the water-soluble polymer PEG in the membranes resulted in hydrophilization and a decrease of the water contact angle. The measured water contact angle values for CA/PEG and CA/PEG/5-Cl8Q membranes were 0°.

The mechanical properties of the electrospun fibrous materials depend on their composition, fiber diameters, fiber alignment, presence of defects, crystallinity degree, etc. [30]. There are different approaches to enhancing the mechanical properties of the fibrous mats; for instance, it is possible to prepare fibrous materials with the desired strength by varying the weight ratios of the two polymers [31] or by the interconnection of fibers [30]. We have proved that an issue of great significance is the direction in which the test specimens are cut, whether parallel (0°) or at a definite angle with respect to the collector rotation direction (45, 90° or others). The obtained results demonstrated that the mats cut in the collector rotation direction (0°) display better mechanical properties compared to those cut at an angle of 90°. Therefore, in the present study, we have performed the mechanical testing of membranes cut in the collector rotation direction. The measurement of the mechanical strength of the obtained membranes showed that CA membranes had the highest values of tensile strength. However, the detected values for the tensile strength and Young’s modulus of the CA nanofibrous membranes were relatively low. This is most probably caused by the semirigid backbone structure of cellulose acetate and due to the fact that the fibers are loosely packed together. It was found that the incorporation of 5-Cl8Q does not alter the mechanical properties of the membranes. However, the incorporation of low-molecular-weight PEG resulted in a decrease of the tensile strength of the membranes. The observed decrease in mechanical properties is in good agreement with the results of Chen et al. [32], who reported lower values regarding the ultimate strength and ultimate strain of all the composite CA/PEG fibers compared to those of CA fibers. They have assumed that the decrease of the mechanical properties of the PEG/CA composite fibers was due to the introduction of PEG, which broke the continuous phase structure of CA. Therefore, the addition of PEG had an unfavorable effect on the tensile properties of the composite fibers. We succeeded in obviating this effect to some extent by using high-molecular-weight polyoxyethylene (MW 100,000) instead of PEG_2000_.

Experiments to determine the release behavior of the bioactive compound from the obtained membranes were performed. The greatest extent of release of 5-Cl8Q was from the CA/PEG/5-Cl8Q membrane. We supposed that the facilitated 5-Cl8Q release was due to the presence of the water-soluble polymer PEG in the polymer membrane.

8-Hydroxyquinoline derivatives display a broad range of biological activities, including antifungal activity against pathogenic fungi causing diseases in humans and in animals [33]. The low MIC values of 5-Cl8Q determined by us against *P. chlamydospora* and *P. aleophilum* [12] give us reason to expect that the incorporation of 5-Cl8Q in fibrous membranes will result in efficient antifungal membranes. Because of the growth characteristics of *P. chlamydospora* and *P. aleophilum* fungi, their development was followed for 96 h. All the membranes containing 5-Cl8Q completely inhibited fungal growth within 96 h; this is due to the fact that the 5-Cl8Q content in the membranes was much higher than the determined MIC of 5-Cl8Q, and, in addition, the release profile of 5-Cl8Q provided sufficient even in the early stages of the experiments.

## 5. Conclusions

Novel micro- and nanofibrous membranes of cellulose acetate and cellulose acetate/PEG containing 5-chloro-8-hydroxyquinolinol (5-Cl8Q) were successfully prepared by electrospinning. The addition of PEG led to the hydrophilization of the membranes and facilitated their wetting. It was demonstrated that the 5-Cl8Q release profile can be modulated by the appropriate selection of the composition of the electrospun membrane. The incorporation of 5-Cl8Q in the membranes imparted a considerable antifungal effect against *P. chlamydospora* and *P. aleophilum* fungi. These features indicate that the obtained novel fibrous membranes are suitable candidates for application in agriculture for plant protection against two main causative agents of esca disease.
